# The deubiquitinating enzyme, ubiquitin‐specific peptidase 50, regulates inflammasome activation by targeting the ASC adaptor protein

**DOI:** 10.1002/1873-3468.12558

**Published:** 2017-01-29

**Authors:** Jae Young Lee, Dongyeob Seo, Jiyeon You, Sehee Chung, Jin Seok Park, Ji‐Hyung Lee, Su Myung Jung, Youn Sook Lee, Seok Hee Park

**Affiliations:** ^1^ Department of Biological SciencesSungkyunkwan UniversitySuwonKorea

**Keywords:** ASC adaptor protein, Inflammasome, USP50 deubiquitinating enzyme

## Abstract

NOD‐like receptor family protein 3 (NLRP3)‐mediated inflammasome activation promotes caspase‐1‐dependent production of interleukin‐1β (IL‐1β) and requires the adaptor protein ASC. Compared with the priming and activation mechanisms of the inflammasome signaling pathway, post‐translational ubiquitination/deubiquitination mechanisms controlling inflammasome activation have not been clearly addressed. We here demonstrate that the deubiquitinating enzyme USP50 binds to the ASC protein and subsequently regulates the inflammasome signaling pathway by deubiquitinating the lysine 63‐linked polyubiquitination of ASC. USP50 knockdown in human THP‐1 cells and mouse bone marrow‐derived macrophages shows a significant decrease in procaspase‐1 cleavage, resulting in a reduced secretion of IL‐1β and interleukin‐18 (IL‐18) upon treatment with NLRP3 stimuli and a reduction in ASC speck formation and oligomerization. Thus, we elucidate a novel regulatory mechanism of the inflammasome signaling pathway mediated by the USP50 deubiquitinating enzyme.

## **Abbreviations**

**AIM2**, absent in melanoma 2

**Alum**, alum crystals

**ASC**, apoptosis‐associated speck‐like protein containing a caspase activation and recruitment domain

**BMDM**, bone marrow‐derived macrophages

**IL‐18**, interleukin‐18

**IL‐1β**, interleukin‐1β

**NIG**, nigericin

**NLRP3**, NOD‐like receptor family protein 3

**TLR**, toll‐like receptor

**TNF**, tumor necrosis factor

**USP50**, ubiquitin‐specific peptidase 50

The innate immune system provokes inflammatory responses to protect cells and tissues from pathogenic infections, cellular damage, and other stressful insults. Initiation of innate immune responses is mediated by pattern recognition receptors (PRRs) which recognize pattern‐ and damage‐associated molecular patterns (PAMPs and DAMPs) 1, 2. NOD‐like receptor family protein 3 (NLRP3) is an important PRR involved in antiviral and antibacterial innate immunity as well as in cellular responses to various proinflammatory stimuli associated with tissue damage 2, 3. Upon activation, NLRP3 forms a multiprotein cytosolic complex, called the inflammasome, together with the ASC protein as an adaptor and procaspase‐1 as an effector 2, 3. Formation of this multiprotein complex eventually results in the autocatalysis and activation of procaspase‐1, which in turn generates biologically active forms of interleukin‐1β (IL‐1β) and interleukin‐18 (IL‐18) through the cleavage of immature proforms 2, 3.

The NOD‐like receptor family protein 3‐mediated inflammasome activation is known to require at least two distinct signals: priming and activation. The priming step requires an increased expression of the NLRP3 and pro‐IL‐1β genes, experimentally triggered by lipopolysaccharide (LPS) treatment 2, 4. The next activation step is the proteolytic processing of pro‐IL‐1β and pro‐IL‐18 by autocatalytic activation of procaspase‐1 upon treatment of NLRP3 stimuli 5. Although a number of studies have reported on the underlying mechanisms of the inflammasome signaling pathway, post‐translational regulation through ubiquitin‐mediated modification of each component of the inflammasome complex is much less characterized 3. Among post‐translational modifications involving covalent ubiquitin chains, deubiquitinating enzymes and E3 ubiquitin ligases were recently implicated as having significant roles in the inflammasome activation. BRCC3 is known to regulate inflammasome activity through deubiquitination of the NLRP3 protein 6. In contrast, A20 has been reported to suppress caspase‐1 activity by restricting K63‐linked ubiquitination of pro‐IL‐1β 7, 8. Ubiquitination and degradation of NLRP3 was also reported to be regulated by the SCF^FBXL2^ E3 ubiquitin ligase 9.

While most studies regarding ubiquitin‐mediated post‐translational modification have focused on regulation of the NLRP3 protein, the ubiquitinating or deubiquitinating mechanisms of other essential components in inflammasome activation such as the ASC adaptor protein are not well understood. The ASC protein induces cytosolic macromolecular aggregates of ASC polymers, called ASC specks, during inflammasome activation 3, 10, 11. A recent study revealed that the linear ubiquitin assembly complex (LUBAC) induces linear ubiquitination of the ASC protein upon treatment of NLRP3 stimuli 12. Also, the ASC protein has been reported to undergo lysine 63 (K63)‐linked polyubiquitination after absent in melanoma 2 (AIM2), another PRR for the inflammasome signal, is activated 13. However, the deubiquitinases involved in modulating ubiquitin‐mediated modification of the ASC protein remain unknown. In this study, we provide experimental evidence that the deubiquitinase, ubiquitin‐specific peptidase 50 (USP50), is a crucial regulator of the ASC protein and demonstrate a role of USP50 in inflammasome activation in mouse bone marrow‐derived macrophages (mBMDMs) as well as human THP‐1 cells.

## Materials and methods

### Plasmids

Full‐length human Flag‐NLRP3 complementary DNA (cDNA) was kindly provided by Je‐Wook Yu (Yonsei University, Korea). After PCR amplification of NLRP3 cDNA, the amplified DNA was subcloned into the *Eco*RV and *Xho*I sites of the pcDNA3‐HA (Invitrogen, Carlsbad, CA, USA), generating the HA‐NLRP3 plasmid. cDNAs encoding human ASC and the procaspase‐1 genes were amplified from human THP‐1 mRNA by PCR and subcloned into the *Eco*RI and *Xho*I sites of the pcDNA3‐Flag (Invitrogen) or pcDNA‐HA vector, respectively, resulting in Flag‐ASC, HA‐ASC, and Flag‐procaspase‐1 plasmids. The cDNA‐encoding mouse pro‐IL‐1β was amplified from mouse BMDM mRNAs by PCR and subcloned into the *Eco*RI and *Xho*I sites of pcDNA3‐Flag, resulting in Flag‐pro‐IL‐1β plasmid. The full‐length cDNA of USP50 was amplified by PCR from the plasmid‐encoding Flag‐HA‐USP50, which was kindly provided by Jaewhan Song (Yonsei University, Korea), and subcloned into the *Eco*RI and *Xho*I sites of pcDNA‐Flag, generating the Flag‐USP50 plasmid. Catalytic inactive mutants [Flag‐USP50(C53S) and Flag‐USP50(H322A)] of the USP50 gene were generated using the QuikChange Mutagenesis kit (Stratagene La Jolla, CA, USA). Wild‐type His‐tagged ubiquitin (His‐Ubi), His‐UbiK48, and His‐UbiK63 were previously described 14. PCR‐generated portions of all constructs were verified by sequencing. Primers for PCR and site‐directed mutagenesis are described in Table S1.

### Cell culture and reagents

Human monocytic THP‐1 cells and human embryonic kidney 293 (HEK293) cells were obtained from the American type culture collection (ATCC). THP‐1 cells were maintained in RPMI‐1640 (HyClone Logan, UT, USA) with 10% heat‐inactivated fetal bovine serum (FBS) and 1% penicillin–streptomycin (Invitrogen). HEK293 cells were maintained in Dulbecco's modified Eagle's medium (DMEM; HyClone) with 10% heat‐inactivated FBS and 1% penicillin–streptomycin at 37 °C in a humidified 5% CO_2_ incubator. Human monocytic THP‐1 cells were differentiated into macrophages with 100 nm phobol 12‐myristate 13‐acetate (PMA, Sigma Aldrich, St. Louis, MO, USA) overnight. BMDMs were obtained by culturing bone marrow cells from 6‐ to 8‐week‐old C57BL/6 mice in MEM alpha medium (Gibco, Waltham, MA, USA) supplemented with 10% heat‐inactivated FBS, 1% penicillin–streptomycin, and 30 ng/mL recombinant murine M‐CSF (Peprotech Rocky Hill, NJ, USA). Animal experiments for the preparation of mouse BMDMs were approved by the Sungkyunkwan University School of Medicine Institutional Animal Care and Use Committee (IACUC). Differentiated THP‐1 cells and mouse BMDMs were primed with 1 μg/mL ultrapure LPS (Sigma) for 4 h and the primed cells were stimulated by 300 μg/mL alum crystals (Alum; Invitrogen, Carlsbad, CA, USA) for 6 h or 10 μm nigericin (NIG; Adipogen, San Diego, CA, USA) for 1 h in six‐well plates. For AIM2 inflammasome activation, differentiated THP‐1 cells were transfected with 1 μg/mL poly (dA : dT) using Lipofectamin 2000 (Invitrogen Carlsbad, CA, USA) for 16 h.

### Transfection of siRNA

To screen for human deubiquitinating enzymes involved in NLRP3 inflammasome activation, the Human ON‐TARGETplus siRNA Library‐Deubiquitinating Enzymes‐SMARTpool (Catalogue #; G‐104705) was obtained from Dharmacon (Lafayette, CO, USA). For transient knockdown of human *USP50* mRNAs, siRNA pools (siUSP50s) targeting *USP50* mRNA were obtained from Dharmacon. PMA‐stimulated THP‐1 cells (1.4 × 10^6^ cells/well) were seeded in six‐well plates and transfected with 40 nm siRNA pools (siUSP50s) for human *USP50* mRNA or the siRNA‐negative control (siCON; Nontargeting pool; Dharmacon) using the Lipofectamin RNAiMAX reagent (Invitrogen). After incubation for 24 h, the culture medium was changed to complete medium. Cells were primed by LPS for 4 h, and subsequently stimulated by Alum and NIG. The siRNA sequences targeting endogenous human *USP50* are described in Table S2.

### Gene knockdown by lentiviruses in mouse BMDMs

*USP50* knockdown in mouse BMDMs were performed by infections of lentiviruses expressing short‐hairpin RNAs specifically targeting *USP50* mRNA. Lentiviral pLKO.1 plasmids expressing shRNAs targeting mouse *USP50* mRNA were purchased from Sigma. In this study, two different shRNAs, sh‐mUSP50#3 (TRCN0000379180) and sh‐mUSP50#4 (TRCN0000374863) were used. The shRNA sequences targeting endogenous mouse *USP50* mRNA are described in Table S2. A lentivirus expressing an siRNA sequence targeting a GFP was used as a negative control. Lentiviruses expressing each shRNA were produced by a lentiviral packaging system from Invitrogen. To generate lentiviruses, HEK293FT cells were transfected with pLKO‐puro lentiviral vectors expressing sh‐mUSP50#3 or sh‐mUSP50#4 in combination with the lentiviral packaging system (Invitrogen), respectively. The culture media containing virus particles was harvested after 48 h and concentrated by the Lenti‐X concentrator (Clontech, Shiga, Japan). The concentrated media was added to differentiated mouse BMDM cells and subsequently incubated for 24 h with polybrene (8 μg/mL) and recombinant murine M‐CSF (30 ng/mL). After incubation, the media was replaced with complete media with M‐CSF. After 48 h, virus‐infected cells were collected, counted, plated, and used for experiments on the next day. The knockdown efficiency was analyzed by quantitative real‐time RT‐PCR (qRT‐PCR). Relative abundance of the transcripts was normalized against *Gapdh* mRNA.

Details of the immunofluorescence, immunoblot (IB), immunoprecipitation, antibodies, ASC oligomerization, measurement of cytokines, RNA extraction and qRT‐PCR, pull‐down and ubiquitination assay by Ni‐nitrilotriacetic acid columns, *in vitro* deubiquitination assay, *in vivo* ubiquitination assay of endogenous ASC protein, and statistical analysis are provided in the Supporting information.

## Results

### Identification of USP50 as a regulator of inflammasome activation through RNAi screening of human deubiquitinating enzymes

To identify the molecular players involved in ubiquitin‐mediated modifications of inflammasome activation, we screened for deubiquitinating enzymes involved in the regulation of NLRP3‐mediated inflammasome activation by using a library containing 99 siRNAs specific for all known human deubiquitinating enzymes (Fig. S1A). Human THP‐1 cells, differentiated by PMA, were respectively transfected with each siRNA in the human deubiquitinating enzyme library. After these cells were treated with LPS for 4 h, followed by NIG treatment for 1 h, we measured secretion of IL‐1β by an enzyme‐linked immunosorbent assay (ELISA). Through three consecutive screenings, we identified two deubiquitinases, A20 (TNFAIP3) and USP50, which caused changes in IL‐1β secretion (Fig. S1B). A20 depletion significantly increased IL‐1β secretion in human THP‐1 cells, indicating that A20 negatively regulates inflammasome activity. These results were consistent with previous reports 7, 8. In contrast, USP50 depletion significantly decreased IL‐1β secretion, suggesting the possibility that USP50 is a positive regulator in inflammasome activation. This was a novel role for USP50, which had previously been reported to be a regulator of the G2/M checkpoint in the cell cycle 15.

### USP50 promotes inflammasome‐mediated cytokine release in human macrophages

**Figure 1:**
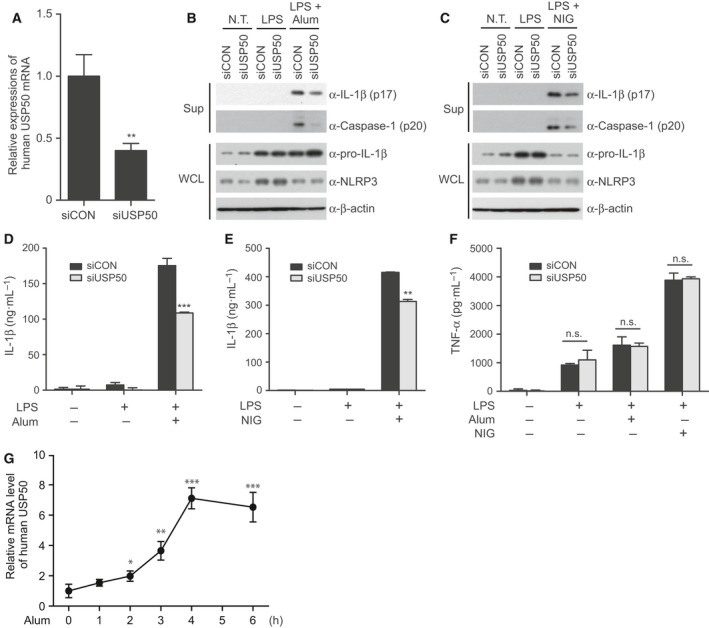
USP50 acts as a positive regulator of inflammasome activation. (A) Human THP‐1 cells were knocked down by an siRNA pool (Dharmacon) targeting *USP50* mRNA (siUSP50). USP50 depletion was confirmed by real‐time RT‐PCR. A nontargeting siRNA pool (siCON), obtained from Dharmacon, was used as a negative control. The data were statistically analyzed by a *t*‐test and show the mean ± SD of three independent experiments. **P < 0.01 compared to the negative control. (B, C) Differentiated THP‐1 cells, knocked down by a negative control siRNA pool or a *USP50*‐specific siRNA pool, were treated with LPS, followed by treatment with Alum (300 μg/mL) for 6 h or NIG (10 μm) for 1 h. Expression of active caspase‐1 (p20) and the mature form of IL‐1β (p17) in culture supernatants (Sup) and WCL were observed by IB analysis. The data in (B) and (C) are representative of at least three independent experiments. (D, E) *USP50‐*knockdown THP‐1 cells or control THP‐1 cells were primed with LPS, followed by Alum (D) or NIG (E) treatment. Secretion of IL‐1β into the culture supernatant was analyzed by ELISA. (F) TNF‐α secretion into the culture supernatants of (D) and (E) were analyzed by ELISA. The data in (D–F) are representative of three independent replicates, statistically analyzed by a *t*‐test and show the mean ± SD. **P < 0.01, ***P < 0.001 compared to control THP‐1 cells treated with NLRP3 stimuli. n.s., not significant. (G) Expression of *USP50* mRNA at the indicated times were analyzed by qRT‐PCR in differentiated THP‐1 cells. The cells were primed with LPS, followed by Alum treatment (300 μg/mL) as indicated. The data were statistically analyzed by a *t*‐test and show the mean ± SD of three independent experiments. *P < 0.05, **P < 0.01, ***P < 0.001 compared to THP‐1 cells untreated with Alum.

To verify the role of USP50 in inflammasome activation, we first generated USP50‐knockdown human THP‐1 cells by using a pool of different siRNAs. Depletion of the *USP50* gene was detected by real‐time RT‐PCR analysis because most commercially available antibodies against the USP50 protein did not detect the expression of endogenous USP50 in our hands (Fig. 1A). To validate whether USP50 depletion affects NLRP3‐mediated inflammasome activation, USP50‐knockdown THP‐1 cells were primed by LPS treatment and subsequently stimulated by Alum or NIG to measure the activation of procaspase‐1 and IL‐1β secretion. IB analysis indicated that the amounts of cleaved caspase‐1 and secreted IL‐1β were significantly decreased in culture media upon treatment of Alum or NIG, compared to wild‐type THP‐1 cells transfected with control siRNAs (Fig. 1B,C). ELISA analysis also showed that IL‐1β secretion is significantly decreased in USP50‐knockdown THP‐1 cells (Fig. 1D,E). In contrast, expressions of pro‐IL‐1β and NLRP3 protein in whole cell lysates (WCL) were not affected by USP50 depletion (Fig. 1B,C). In contrast to the significant reduction in IL‐1β secretion upon USP50 depletion, tumor necrosis factor (TNF)‐α release in culture media was not affected by USP50 knockdown (Fig. 1F). Because the expression of TNF‐α is regulated by the toll‐like receptor (TLR) signaling pathway triggered by LPS treatment 16, our results indicate that USP50 is not involved in the priming step, but is involved in the activation step of inflammasome signaling by NLRP3 stimuli such as Alum and NIG treatment. Additionally, following pretreatment of human THP‐1 cells by LPS, *USP50* mRNA expression showed a time‐dependent increase upon Alum treatment (Fig. 1G), suggesting the possibility that USP50 might be a target protein induced by danger signals. Therefore, our results suggest that USP50 is required for effective activation of the NLRP3 inflammasome and IL‐1β secretion upon stimulation of the NLRP3 ligands.

### USP50 binds to the ASC adaptor protein and is required for NLRP3‐induced ASC speck formation and oligomerization

**Figure 2:**
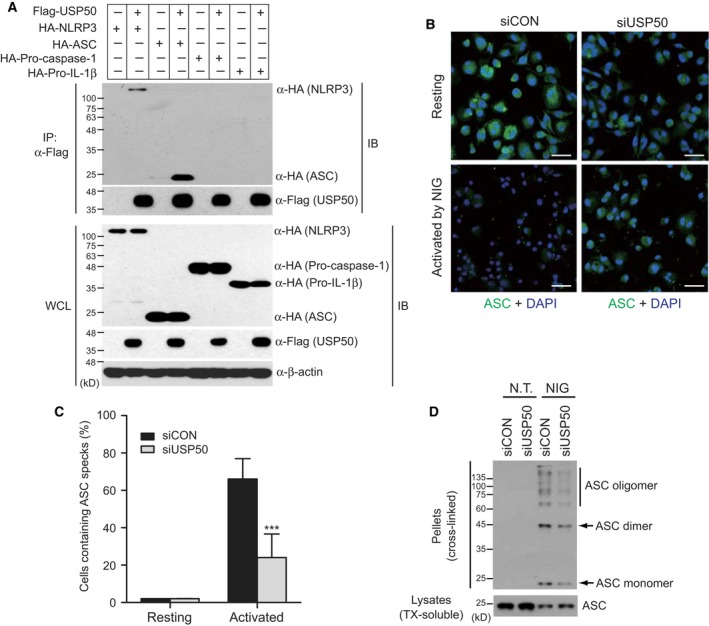
USP50 binds to the ASC protein and is required for ASC speck formation and oligomerization. (A) Coimmunoprecipitation assays to demonstrate the interaction of USP50 with the ASC protein. Plasmids encoding HA‐NLRP3, HA‐ASC, HA‐HA‐procasapase‐1 or HA‐pro‐IL‐1beta were transfected into HEK293 cells in the absence or presence of Flag‐tagged USP50 as indicated. Cell lysates were immunoprecipitated using Flag antibody. WCL and immunoprecipitates (IP) were immunoblotted with the indicated antibodies. The data are representative of at least three independent experiments. (B) THP‐1 cells, knocked down by siCON and siUSP50 pools, were untreated (upper panel, Resting) or treated (lower panel, Activated) with NIG (10 μm) for 1 h and subsequently immunostained by anti‐ASC and Alexa Fluor 488‐conjugated secondary antibodies, together with DAPI for nuclear stain. Images were acquired by laser scanning confocal microscopy. The images were merged, with ASC (green) and nucleus (blue). Scale bars, 50 μm. The data are representative of at least three independent experiments. (C) ASC speck‐forming cells were scored after NIG stimulation in two independent experiments. ASC specks were counted in five random areas of each image in duplicate experiments and described as a percentage of ASC specks for total cell nuclei. In each region, at least 200 cells were blindly counted. The data were statistically analyzed by a *t*‐test and show the mean ± SD. ***P < 0.001 compared to THP‐1 cells treated with a control siRNA pool. (D) ASC aggregation was analyzed by IB analysis in control THP‐1 and USP50‐knockdown THP‐1 cells. IB analysis for the ASC oligomerization assay was performed by anti‐ASC antibody in the pellets (cross‐linked by disuccinimidyl suberate) and soluble lysates (treated with TX) of THP‐1 cells, treated with or without NIG. The data are representative of three independent experiments. TX, Triton X‐100.

Our findings that USP50 is needed for inflammasome activation prompted us to examine the binding partners between USP50 and the NLRP3 inflammasome complex. Plasmids encoding HA‐NLRP3, HA‐ASC, HA‐procaspase‐1, and HA‐pro‐IL‐1β were transiently transfected into HEK293 human embryonic kidney cells in the absence or presence of Flag‐USP50, respectively, as indicated (Fig. 2A). Coimmunoprecipitation assays indicated that USP50 strongly binds to the ASC adaptor protein, although it also interacted weakly with NLRP3 (Fig. 2A). In contrast, USP50 did not bind to procaspase‐1 and pro‐IL‐1β (Fig. 2A). The strong association between USP50 and the ASC protein suggests that the ASC protein may be a target of USP50.

The ASC protein, which acts as an adaptor in the inflammasome complex, is known to assemble into large protein complexes, termed ASC specks, following inflammasome activation 17, 18. Therefore, ASC speck formation can be used as a readout for inflammasome activation. Based on the interaction between USP50 and ASC protein, we next investigated whether USP50 knockdown affects inflammasome activation by analyzing the formation of ASC specks. USP50‐knockdown THP‐1 cells were treated with NIG and stained with an antibody against endogenous ASC, according to a previously reported protocol 19. Compared with control THP‐1 cells, the amounts of ASC specks induced by NIG treatment were significantly reduced in USP50‐knockdown THP‐1 cells (Fig. 2B). ASC specks were counted in five random areas of each image for triplicate experiments and described as the percentage of ASC specks in total cell nuclei (Fig. 2C). We next examined ASC oligomerization, a common event associated with inflammasome activation, in USP50‐knockdown THP‐1 cells. After USP50‐knockdown and control THP‐1 cells were treated with or without NIG, we prepared the ASC pyroptosome, a complex of monomers, dimers, and oligomers, by applying disuccinimidyl suberate to cytosolic fractions of cell lysates and subsequently performed IB assays. ASC oligomerization was almost undetectable in both control and USP50‐knockdown THP‐1 cells in the absence of NIG (Fig. 2D). This oligomerization was obviously increased in control THP‐1 cells upon NIG treatment, but significantly reduced in USP50‐knockdown THP‐1 cells (Fig. 2D). These results provide robust evidence that USP50 is required for the effective formation of ASC specks as well as ASC oligomerization through interaction with the ASC protein.

### USP50 is crucial for the activation of the NLRP3 inflammasome in mouse bone marrow‐derived macrophages (BMDMs)

To exclude the possibility that the requirement for USP50 is specific for human THP‐1 cells, we generated two USP50‐knockdown mouse BMDMs through the infection of lentiviruses independently expressing different short hairpin RNAs (shRNAs) against mouse *USP50* mRNA. As a negative control, lentiviruses expressing shRNA against the mRNA of GFP were infected into mouse BMDMs. Depletion of USP50 mRNA in mouse BMDMs was confirmed by quantitative real‐time RT‐PCR (Fig. S2A). USP50‐knockdown mouse BMDMs were primed by LPS treatment and subsequently activated by Alum and NIG, respectively. ELISA assays indicated that IL‐1β secretion into culture media from USP50‐knockdown mouse BMDMs is significantly decreased, compared to control cells (Fig. S2B,C). In addition, secretion of IL‐18, another target cytokine of the inflammasome signaling pathway, was also reduced (Fig. S2D,E). Furthermore, IB analysis revealed that the amounts of cleaved caspase‐1 and secreted IL‐1β were significantly decreased in culture media upon treatment of Alum or NIG in USP50‐knockdown mouse BMDMs, compared to control mouse BMDMs (Fig. S3A,B). In contrast, LPS‐induced TNF‐α release was unaffected by USP50 depletion in mouse BMDMs (Fig. S2F). These results were similar to the results obtained from USP50‐knockdown human THP‐1 cells. Therefore, USP50 appears to act as a positive regulator in the NLRP3‐mediated inflammasome signaling pathway and to be involved in the activation step of inflammasome activation through interacting with the ASC protein, but not the priming step.

### USP50 deubiquitinates the ASC protein

**Figure 3:**
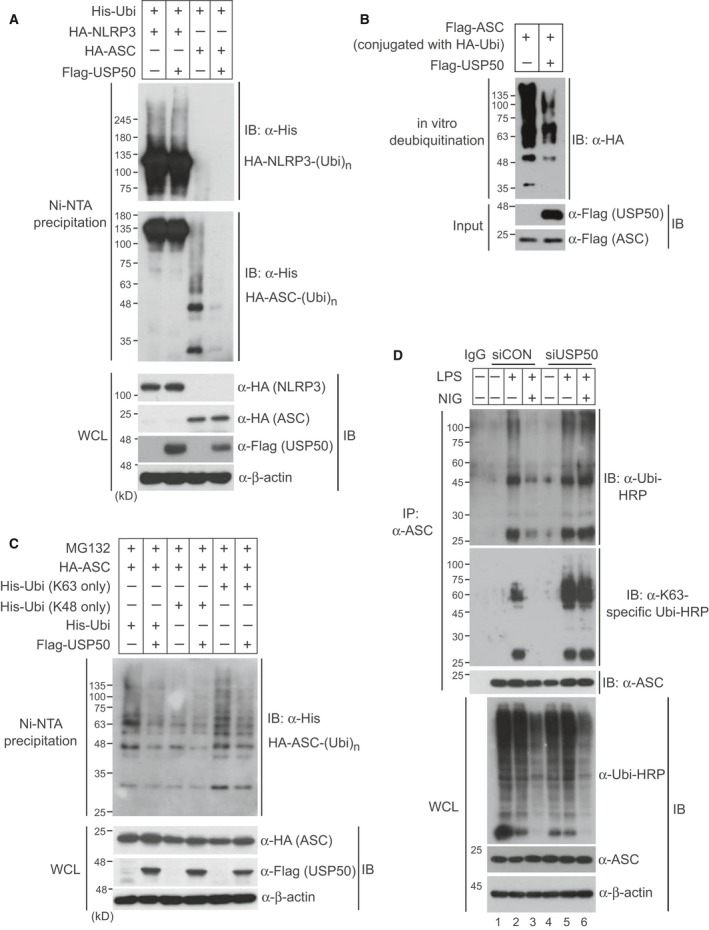
USP50 directly deubiquitinates K63‐linked polyubiquitination of the ASC protein. (A) A plasmid encoding HA‐NLRP3 or HA‐ASC was cotransfected with wild‐type His‐Ubi into HEK293 cells in the absence or presence of Flag‐USP50. After cells were lysed under the denaturing conditions of 6 m guanidine‐HCl, Ni‐nitrilotriacetic acid‐mediated pull‐down assays were performed and the precipitates were subsequently separated with 6% polyacrylamide gel (upper panel) for NLRP3 or 12% polyacrylamide gel (lower panel) for ASC. Separated samples were analyzed by IB with the indicated antibodies. (B) For an *in vitro* deubiquitination assay, Flag‐ASC protein conjugated with HA‐ubiquitin (HA‐Ubi) was eluted from HEK293 cells which were cotransfected with Flag‐ASC and HA‐Ubi plasmids. Also, Flag‐USP50 protein was eluted from HEK293 cells transfected with the Flag‐USP50 plasmid. The reactions were performed in the indicated combinations and were analyzed by IB with the indicated antibodies. (C) Plasmids encoding His‐Ubi or ubiquitin mutants (K48 only and K63 only) were cotransfected with the HA‐ASC plasmid into HEK293 cells in the absence or presence of Flag‐USP50. After cells were treated with the proteasome inhibitor MG132 for 6 h, Ni‐nitrilotriacetic acid‐mediated pull‐down assays were performed and analyzed by immunoblotting with the indicated antibodies. (D) For *in vivo* ubiquitination assay of endogenous ASC protein, USP50‐knockdown and control THP‐1 cells were primed with LPS and treated with or without NIG. Cell lysates were immunoprecipitated with anti‐ASC antibody under 1% SDS denaturating condition and subsequently immunoblotted with anti‐ubiquitin‐HRP and anti‐K63 linkage‐specific ubiquitin‐HRP antibodies. WCL were immunoblotted with the indicated antibodies. All data for IB analysis in this figure are representative of at least three independent experiments.

Although NLRP3 was known to be modified by the deubiquitinating enzyme BRCC3 6, the deubiquitinating enzyme regulating the ubiquitination pattern of the ASC adaptor protein remains unknown. To understand the molecular mechanism of action of the USP50‐ASC axis on the regulation of inflammasome activation, we examined ubiquitination of the ASC protein as well as NLRP3 protein in the presence or absence of USP50. After plasmids encoding His‐tagged ubiquitin (His‐Ubi), HA‐NLRP3, or HA‐ASC were transiently transfected into HEK293 cells with or without Flag‐USP50, NLRP3 and ASC ubiquitination was examined by Ni‐nitrilotriacetic acid agarose‐mediated pull‐down experiments (Fig. 3A). To exclude nonspecific binding to ubiquitin, the pull‐down assays were performed under denaturing conditions of 6 m guanidine‐HCl. Expression of USP50 significantly inhibited polyubiquitination of the ASC protein, but not the NLRP3 protein (Fig. 3A). These results demonstrate that among the inflammasome components, ASC protein is a major target of USP50.

To further confirm our finding that USP50 deubiquitinates the ASC protein, we performed *in vitro* deubiquitination assays. After HEK293 cells were cotransfected with Flag‐tagged ASC (Flag‐ASC) and HA‐tagged ubiquitin (HA‐Ubi) plasmids, Flag‐ASC proteins conjugated with HA‐Ubi were precipitated with Flag antibody‐conjugated beads and then eluted by Flag peptides. Flag‐USP50 proteins were also prepared through the same protocol. After Flag‐ASC proteins conjugated with HA‐Ubi were reacted with or without eluted Flag‐USP50 proteins *in vitro*, the ubiquitination patterns of Flag‐ASC proteins were detected by IB analysis with anti‐HA antibody. Ubiquitination of Flag‐ASC proteins was significantly decreased in the presence of Flag‐USP50 protein, indicating that USP50 directly deubiquitinates the polyubiquitination of the ASC protein (Fig. 3B).

Next, we investigated which polyubiquitination pattern of the ASC protein is regulated by USP50. Plasmids encoding wild‐type ubiquitin (His‐Ubi), the K48 ubiquitin mutant (His‐Ubi‐K48) in which six lysine residues except for lysine 48 are substituted into arginines, and the K63 ubiquitin mutant (His‐Ubi‐K63) in which only K63 is left intact, were transfected into HEK293 cells with HA‐ASC in the absence or presence of Flag‐USP50. The cells were treated with the proteasome inhibitor MG132 to prevent degradation of the ASC protein. USP50 specifically decreased K63‐linked polyubiquitination of ASC, similar to the reduction in wild‐type ubiquitin‐linked polyubiquitination, but did not affect K48‐linked polyubiquitination (Fig. 3C).

We subsequently examined whether polyubiquitination of endogenous ASC protein is regulated by USP50. After USP50‐knockdown and control THP‐1 cells were treated with LPS for 4 h, followed by NIG treatment for 1 h, endogenous ASC protein was immunoprecipitated with anti‐ASC antibody and subsequently immunoblotted with anti‐ubiquitin‐horseradish peroxidase (HRP). To exclude nonspecific binding to ubiquitin, the immunoprecipitation assays were performed under the denaturating conditions of 1% SDS and the transferred membranes were denaturated by 6 m guanidine buffer. The increased polyubiquitination of endogenous ASC protein upon LPS treatment was significantly decreased by NIG treatment in control THP‐1 cells (Fig. 3D, lane 2 and 3). This reduction upon NIG treatment was not observed in USP50‐knockdown THP‐1 cells (Fig. 3D, lane 3 and 6). IB analysis using an antibody against K63 linkage‐specific ubiquitin provided support that endogenous ASC protein is polyubiquitinated through the K63‐linked pattern and regulated by USP50 (Fig. 3D). Therefore, these results show that the binding of USP50 with ASC protein to deubiquitinate K63‐linked polyubiquitination is a crucial step for inflammasome activation.

**Figure 4:**
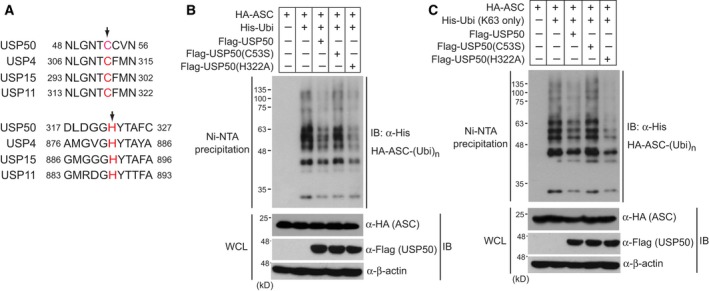
Catalytic inactive mutant of USP50 does not inhibit K63‐linked polyubiquitination of the ASC protein. (A) Putative amino acid residues critical for the deubiquitinating enzyme activity of USP50. The alignment of amino acid sequences of USP50 and other USP proteins shows that the Cys 53 and His 322 residues of USP50 are highly conserved. Arrow indicates the conserved residues tested in this experiment. (B) Plasmids encoding wild‐type His‐Ubi and HA‐ASC were cotransfected with plasmids encoding wild‐type Flag‐USP50, the Flag‐USP50(C53S) mutant or Flag‐USP50 (H322A) mutant into HEK293 cells. After cells were lysed under the denaturing conditions of 6 m guanidine‐HCl, Ni‐nitrilotriacetic acid‐mediated pull‐down assays were performed and analyzed by immunoblotting with the indicated antibodies. (C) Plasmids encoding His‐tagged K63 only ubiquitin mutant [His‐Ubi (K63 only)] and HA‐ASC were cotransfected with a plasmid‐encoding Flag‐USP50 or the above USP50 mutants into HEK293 cells. Ni‐nitrilotriacetic acid pull‐down assays were performed and analyzed by immunoblotting with the indicated antibodies. The data for IB analysis are representative of at least three independent experiments.

To further support the deubiquitinating activity of USP50 on the ASC protein, we generated catalytically inactive USP50 mutants and examined whether these mutants could not decrease polyubiquitinations of the ASC protein. The deubiquitinating enzymes, belonging to the USP family, show a high degree of amino acid homology, predominantly in the regions known as the N‐terminal cysteine (Cys) box and the C‐terminal histidine (His) box 20. These regions surround the catalytic Cys and His residues which have been reported to be essential for the deubiquitinating activity of USP family members 21 (Fig. 4A). Based on homology, we generated two USP50 mutants, in which a Cys residue (amino acid number 53) and His residue (amino acid number 322) were substituted into serine and alanine, respectively. After plasmids encoding these USP50 mutants and wild‐type USP50 were transfected into HEK293 cells together with HA‐ASC and His‐Ubi, Ni‐nitrilotriacetic acid pull‐down and IB analysis were performed. Wild‐type USP50 and the Flag‐USP50(H322A) mutant significantly deubiquitinated the ASC protein (Fig. 4B). However, the Flag‐USP50(C53S) mutant, did not affect polyubiquitination of the ASC protein (Fig. 4B), indicating that the Cys residue at position 53 is critical for the catalytic activity of USP50 deubiquitination of the ASC protein. These findings were further confirmed by experimental results showing that the Flag‐USP50(C53S) mutant did not reduce K63‐linked polyubiquitination of ASC protein (Fig. 4C).

Next, we examined whether USP50 is required for the activation of other inflammasomes that also trigger K63‐linked polyubiquitination of ASC, such as the AIM2 inflammasome. AIM2 is an inflammasome receptor for double‐stranded DNA (dsDNA) with a pyrin domain, and the ASC protein serves as a bridge between procaspase‐1 and PRRs through its caspase recruitment domain (CARD) and pyrin domains 3, 22. After USP50‐knockdown and control THP‐1 cells were transfected with 1 μg poly (dA : dT), experimentally used as a ligand for AIM2 inflammasome 22, 23, IL‐1β secretion was analyzed by ELISA. Similar with the results obtained with NLRP3 ligands (Fig. 1D,E; Fig. S2), IL‐1β secretion was significantly decreased in USP50‐knockdown THP‐1 cells, compared to control THP‐1 cells (Fig. S4A). In contrast, TNF‐α release in culture media was not affected by USP depletion (Fig. S4B). These results indicate that USP50 is involved in both AIM2 and NLRP3 inflammasome activation by targeting the common adaptor ASC protein. Therefore, our findings demonstrate that USP50 regulates the inflammasome signaling pathway by deubiquitinating K63‐linked polyubiquitination of the ASC adaptor protein.

## Discussion

The NLRP3 inflammasome pathway acts as a multiprotein signaling platform in mediating inflammatory responses to clear infected pathogens and initiate wound healing after tissue damage 3, 24. Therefore, inappropriate activation of this pathway causes deleterious inflammatory diseases through inordinate cytokine release, emphasizing that a well‐balanced inflammasome response is crucial for maintaining tissue homeostasis 24, 25. In this respect, it is worth exploring the regulatory mechanisms of inflammasome activation mediated by modifications of inflammasome components. Herein, we have uncovered a novel function of the deubiquitinating enzyme USP50 in NLRP3‐mediated inflammasome activation through targeting the ASC protein.

Although a number of studies reveal that the regulation of covalent conjugation of ubiquitin molecules to target proteins by specific E3 ubiquitin ligases or deubiquitinases contributes to protein stability and protein–protein interaction in innate immunity 26, 27, the regulatory mechanisms of inflammasome activation by the ubiquitination/deubiquitination process has not been addressed to the same extent as other innate immune signaling pathways. The deubiquitinase BRCC3 was shown to regulate inflammasome activity through targeting the NLRP3 protein as a substrate 6, whereas A20, an ubiquitin‐editing enzyme, was found to suppress inflammasome activity by restricting the ubiquitination of lysine 133 of pro‐IL‐1β 8. Although these recent reports suggest the importance of deubiquitination in regulating inflammasome components, the deubiquitinating enzymes that act on the ASC adaptor protein remain unknown. Therefore, this is the first report demonstrating that the ASC protein is a substrate of the deubiquitinating enzyme USP50.

The USP50 protein was previously described to be associated with Hsp90 and to control the protein level of Wee1, an essential component of G2/M cell cycle arrest 15. In this study, we demonstrate a role of USP50 in the inflammasome activation as a positive regulator. Depletion of USP50 caused a significant decrease in ASC oligomerization and ASC specks upon treatment of NLRP3 ligands after the priming step, resulting in the reduction of inflammasome activity. The decreased activity of the inflammasome eventually inhibited IL‐1β and IL‐18 secretion in both human THP‐1 macrophages and mouse BMDMs. In contrast, TNF‐α secretion induced by LPS treatment was not affected by USP50 depletion, indicating that USP50 is not required for the priming step.

In addition, our Ni‐nitrilotriacetic acid pull‐down, *in vitro* deubiquitination, and *in vivo* ubiquitination assays strongly indicate that USP50 deubiquitinates K63‐linked polyubiquitination of the ASC protein (Fig. 3). K63‐linked polyubiquitination to target proteins is generally recognized to be necessary for signal transduction through protein–protein interaction 26, 27. K63‐linked polyubiquitination of the ASC protein was observed in AIM2 inflammasome activation triggered by double‐stranded DNA in the form of poly (dA : dT) and this K63‐linked polyubiquitination of the ASC protein eventually recruited p62 which assisted delivery of the AIM2 inflammasome to the autophagosome 13. Another recent study revealed that TNFR‐associated factor 3 (TRAF3) is a direct E3 ubiquitin ligase promoting K63‐linked polyubiquitination of the ASC protein 28. In contrast, our Ni‐nitrilotriacetic acid pull‐down and *in vivo* ubiqutination assays under denaturing conditions also revealed that K63‐linked polyubiquitination of endogenous ASC protein is removed by the deubiquitinating enzyme USP50. Taken together with our results that USP50 depletion decreases the formation of ASC specks as well as the secretion of IL‐lβ, our findings strongly suggest the possibility that removal of these polyubiquitin chains from ASC protein by USP50 is a crucial step for inflammasome activation. This speculation is supported by the finding that USP50 expression is upregulated upon treatment of NLRP3 ligands. That is, increased USP50 may initiate the formation of the inflammasome complex by binding to the ASC protein and inducing its deubiquitination.

In conclusion, we here demonstrate a novel regulatory mechanism regarding the ASC protein which is deubiquitinated by USP50 and provide experimental evidence emphasizing the importance of a regulatory mechanism mediated by ubiquitination/deubiquitination in inflammasome activation. Further studies on the dynamics between the E3 ubiquitin ligases and USP50 deubiquitinating enzyme that act on the ASC protein may shed light on additional regulatory mechanisms of the inflammasome activation by danger signals.

## Author contributions

JYL designed the research, did the experimental work, analyzed data, wrote the manuscript, participated in the study, and coordinated the study; DS, JY, SC, JSP, J‐HL, and SMJ performed the screening of deubiquitinating enzymes and provided technical assistance; YSL participated in the study and coordinated the study; SHP designed and conceptualized the research, supervised the experimental work, analyzed data, and wrote the manuscript.

## Supporting information


**Fig. S1.** Identification of the deubiquitinating enzymes (DUBs) that regulates NLRP3inflammasome signaling.
**Fig. S2.** USP50 depletion in mouse BMDMs reduces NLRP3 inflammasome activation.
**Fig. S3.** USP50 depletion in mouse BMDMs reduces the secretion of IL‐1β.
**Fig. S4.** USP50 is required for the activation of AIM2 inflammasome.
**Table S1.** Primer sequences used to construct plasmids in this study.
**Table S2.** List of siRNA sequences used in this study.
**Table S3.** Primer for real‐time RT‐PCR used in this study.
