# Supplementary material for: The deubiquitinating enzyme, ubiquitin‐specific peptidase 50, regulates inflammasome activation by targeting the ASC adaptor protein
Source: FEBS Lett. 2017 Jan 29;591(3):479–90. doi: 10.1002/1873-3468.12558 (PMC5324553; doi:10.1002/1873-3468.12558)
Supplement: Supplementary file 1 — Fig. S1. Identification of the deubiquitinating enzymes (DUBs) that regulates NLRP3inflammasome signaling. Fig. S2. USP50 depletion in mouse BMDMs reduces NLRP3 inflammasome activation. Fig. S3. USP50 depletion in mouse BMDMs reduces the secretion of IL‐1β. Fig. S4. USP50 is required for the activation of AIM2 inflammasome. Table S1. Primer sequences used to construct plasmids in this study. Table S2. List of siRNA sequences used in this study. Table S3. Primer for real‐time RT‐PCR used in this study. [file FEB2-591-479-s001.pdf]

Supporting information

The deubiquitinating enzyme USP50 regulates inflammasome activation through targeting the ASC adaptor protein

Jae Young Lee, Dongyeob Seo, Jiyeon You, Sehee Chung, Jin Seok Park, Ji-Hyung Lee, Su Myung Jung, Youn Sook Lee, Seok Hee Park

Supporting information includes

1. Materials and methods:
Immnofluorescence, immunoblot, immunoprecipitation, antibodies, ASC oligomerization, measurement of cytokines, RNA extraction and quantitative real-time PCR (qRT-PCR), pull-down and ubiquitination assay by Ni-NTA columns, *in vitro* deubiquitination assay, *in vivo* ubiquitination assay of endogenous ASC protein and statistical analysis

2. Supplementary Figures:
Supplementary Fig. 1, Supplementary Fig. 2, Supplementary Fig. 3 and Supplementary Fig. 4

3. Supplementary Tables
Supplementary Table 1-3

1. Materials and methods

Immunofluorescence

Briefly, human THP-1 cells, stimulated on coverslips, were washed with PBS twice and fixed in cold methanol for 7 min, followed by blocking with 5 % BSA in PBS and incubation with primary antibodies at room temperature for 3 h. Anti-ASC (sc-22514R , Santa Cruz, 1:100) antibody was used to detect ASC specks. After five washes with PBS, coverslips were incubated at room temperature for 3 h with Alexa Fluor$^{@}$-488-conjugated goat anti-rabbit IgG (Invitrogen, 1:100). All coverslips were stained with DAPI (Santa Cruz) and mounted on glass slides. A laser-scanning confocal microscope (CarlZeiss LSM700) was used to image the fluorescent samples.

Immunoblot, immunoprecipitation and antibodies

Immunoblot analysis was performed as previously described [14]. Briefly, cells were lysed in lysis buffer (1 % Triton X-100, 20 mM Hepes at pH 7.4, 150 mM NaCl, 12.5 mM β-glycerol phosphate, 1.5 mM $MgCl_2$, 10 mM NaF, 2 mM DTT, 1 mM NaOV, 2 mM EGTA, 1mM PMSF, protein inhibitor cocktail). Proteins from cell-free supernatants were precipitated by methanol/chloroform precipitation. Protein extracts and supernatant samples were separated by SDS-PAGE, transferred to a PVDF membrane filter (Millipore), and subjected to immunoblot analysis. The following antibodies were used for immunoblot analysis: mouse anti-HA (16B12, Covance), anti-HA-HRP (12013819001, Roche), and human anti-active-caspase-1 p20 (#4199S, cell signaling). Mouse anti-Flag (F3165), rabbit anti-Flag (F7425) and mouse anti-β-actin (A5316) were obtained from Sigma. Human anti-IL-1β (sc-1250), human anti-caspase-1 (sc-515) and anti-ASC (sc-22514-R) were obtained from Santa Cruz. Anti-NLRP3 (AG-20B-0014) was obtained from Adipogen. For immunoprecipitation, lysates

were incubated with protein G agarose beads (Genedepot) at 4 ºC for 12 h. Immunocomplexes were washed twice with lysis buffer and separated from the beads by adding 2x sample buffer and boiling. Immunoblot analysis was performed using the indicated antibodies.

ASC oligomerization

ASC oligomerization was basically carried out as in the previously described protocol [15]. Pellets from whole-cell lysates were cross-linked with disuccinimidyl suberate (DSS, Sigma Aldrich) for 40 min at 37 ºC. The reaction was stopped by the addition of quenching solution at a final concentration of 20 mM Tris-HCl (pH 7.5) for 10 min at room temperature. Samples were then fractionated by SDS-PAGE with 12 % polyacrylamide gel and immunoblotted with anti-ASC antibody.

Measurement of Cytokines

Cytokines were measured from cell culture supernatants using specific ELISA kits according to the manufacturer's instructions. Secretions of human and mouse IL-1β were analyzed using ELISA kits purchased from BD Biosciences. ELISA kits for human and mouse TNF-α and mouse IL-18 were purchased from eBiosciences and R&D systems, respectively. To examine the effects of USP50 on AIM2-mediated inflammasome activation, secretion of IL-1β and TNF-α into culture media were analyzed by ELISA 16 h after transfection of 1 μg/ml poly(dA:dT) into human THP-1 cells. All reactions were independently repeated at least three times to ensure reproducibility and values were calculated on the basis of a standard curve constructed for each assay.

RNA extraction and quantitative real-time RT-PCR (qRT-PCR)

Total RNA was extracted from cells using the TRIzol reagent (Invitrogen). Oligo-dT and PrimeScript Reverse Transcriptase (TaKaRa) was used for reverse transcription. For qRT-PCR of *USP50* mRNA, an iCycler real-time PCR machine and iQ SYBR Green Supermix (Bio-Rad) were used for real-time detection of PCR products under the following conditions: 40 cycles of 95 °C for 15 sec and 60 °C for 45 sec. The analysis of the expression of *USP50* and *Gapdh* genes was performed using the software provided by Bio-Rad. Primer sequences of the *USP50* and *Gapdh* genes are described in Supplementary Table 3. All qRT-PCR reactions were independently repeated at least three times to ensure reproducibility.

Pull-down and ubiquitination assay by Ni-NTA columns

Ni-NTA-mediated pull-down assays were performed as previously described [14]. Briefly, cells were collected in cold PBS buffer containing 5 mM NEM. Cells were resuspended in binding buffer (6 M guanidine HCl, 0.1 M Na_2HPO_4, 0.1 M NaH_2PO_4, 0.01 M Tris (pH 8.0), 10 mM β-mercaptoethanol, 5 mM NEM, 5 mM imidazole) and incubated with Ni-NTA agarose resin (Qiagen) at 4 °C overnight. The Ni-NTA agarose resin was sequentially washed with buffer A (6 M guanidine HCl, 0.1 M Na_2HPO_4, 0.1 M NaH_2PO_4, 0.01 M Tris (pH 8.0), 10 mM β-mercaptoethanol), buffer B (8 M Urea, 0.1 M Na_2HPO_4, 0.1 M NaH_2PO_4, 0.01 M Tris (pH 8.0), 10 mM β-mercaptoethanol), buffer C (8 M Urea, 0.1 M Na_2HPO_4, 0.1 M NaH_2PO_4, 0.01 M Tris (pH 6.3), 10 mM β-mercaptoethanol, 0.2 % Triton X-100), and buffer D (8 M Urea, 0.1 M Na_2HPO_4, 0.1 M NaH_2PO_4, 0.01 M Tris (pH 6.3), 10 mM β-mercaptoethanol, 0.1 % Triton X-100). The resulting Ni-NTA agarose resin was suspended in elution buffer and loaded onto SDS-PAGE gels. Immunoblotting was performed using the indicated antibodies. Expression of the β-actin protein was used as a loading control.

***In vitro* deubiquitination assay**

To perform *in vitro* deubiquitination assay, HA-ubiquitin conjugated Flag-ASC proteins was generated from HEK293 cells, which were transiently expressing Flag-ASC and HA-ubiquitin plasmids, by immunoprecipitation with anti-Flag® M2 affinity Gel (Sigma Aldrich, A2220) and elution with Flag® peptide (Sigma Aldrich, F3290). Flag-USP50 proteins were also obtained from HEK293 cells through the same protocol and subsequently used as the deubiquitinating enzyme. The eluted Flag-ASC was added to a deubiquitination reaction (40 mM Tris-HCl pH 7.6, 50 mM NaCl, 5 mM $MgCl_2$, 5 mM DTT, 2 mM ATP) in the absence or presence of eluted Flag-USP50. Reactions were incubated at 37 °C for 6 hours, terminated by the addition of 4X SDS sample buffer followed by a heat inactivation with boiling. Immunoblotting was performed using anti-HA antibody to detect HA-ubiquitin-conjugated ASC proteins.

***In vivo* ubiquitination assay of endogenous ASC protein**

Differentiated human THP-1 cells were knockdowned by control siRNA and an USP50-specific siRNA pool (siUSP50s). USP50-knockdown and control THP-1 cells were primed with 1 μg/ml LPS for 4 h and subsequently stimulated by 10 μM nigericin for 1 h. Cells were harvested in 1 ml PBS containing 5 mM N-ethyl maleimide (NEM). Dissociation of non-covalent protein interactions and dilution of samples were previously described [14]. For immunoprecipitation, cell lysates were precleared with 1 μg IgG antibody bound to protein G agarose beads (GenDEPOT) for 12 h at 4 °C. The precleared lysates were immunoprecipitated with 1 μg anti-ASC antibody (AG-25B-0006-C100, Adipogen) or control IgG antibody bound to protein G agarose beads. The beads were washed three times with lysis buffer and immunoprecipitates were separated from the beads by adding 2X sample buffer. After boiling, the immunoprecipitates were fractioned by SDS-PAGE. The separated immunoprecipitates were transferred onto PVDF membranes. The membranes were

denaturated by 6 M guanidine buffer (TBS contacting 6 M guanidine chloride, 20 mM Tris-HCl pH 7.5 and 5mM β-mercaptoethanol) at 4 °C for 30 min. After denaturation, membranes were washed by TBST three times. The membranes were blocked with 5 % BSA and incubated with anti-FK2-HRP antibody (BML-PW8810, Enzo lifesciences) and anti-K63-linkage-specific-HRP antibody (BML-PW0605, Enzo lifesciences).

Statistical analysis

All experiments have at least three biological replicates. Results are expressed as mean ± SD. Statistical analyses were made with an unpaired *t*-test for two groups using Prism software (GraphPad). A P value <0.05 was considered as a statistically significant difference. Differences with $P<0.05$ are annotated as *, $P<0.01$ are annotated as ** and $P<0.001$ as ***.

2. Supplementary Figures

Supplementary Fig. 1

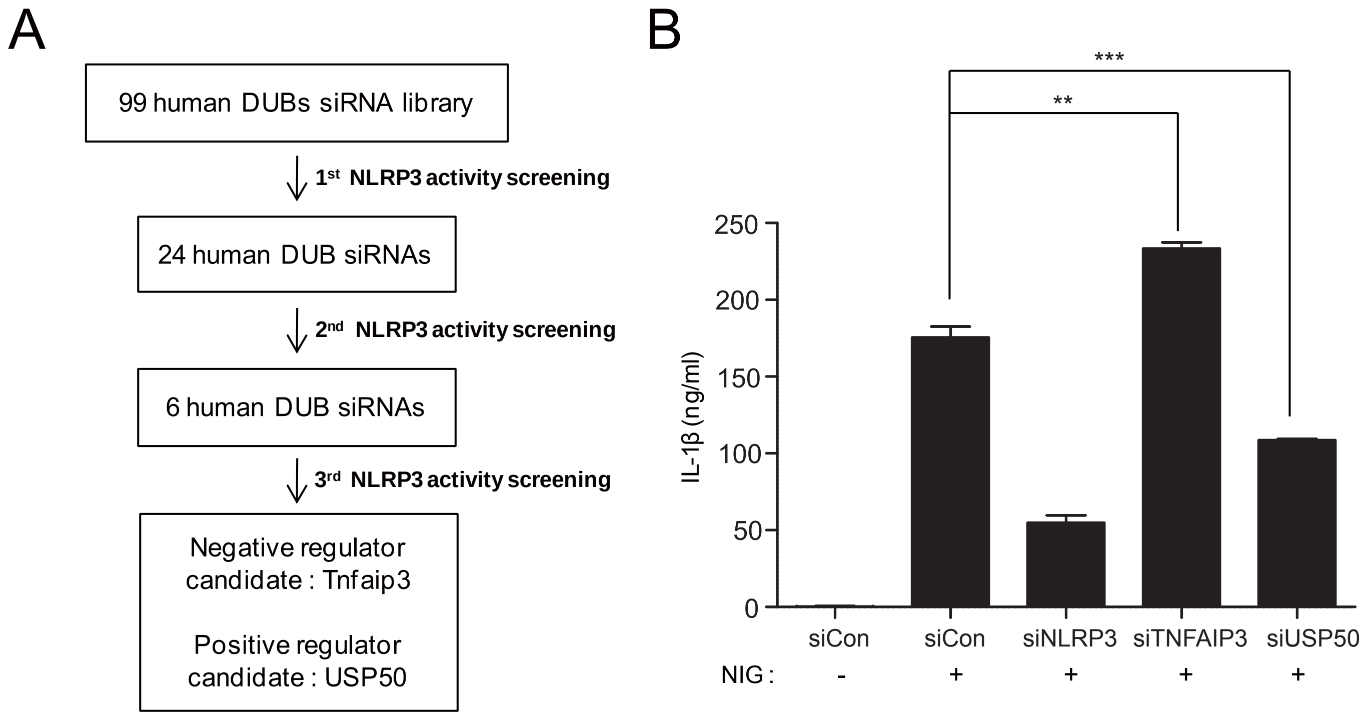

Supplemenatary Fig. 1. Identification of the deubiquitinating enzymes (DUBs) that regulates NLRP3 inflammasome signaling
(**A**) The screening strategies to identify the DUBs regulating NLRP3 inflammasome signaling in human THP-1 macrophages. (**B**) THP-1 cells, knockdowned by the indicated DUB-specific siRNAs, were treated with LPS for 4 h, followed by nigericin (NIG, 10 μM) for 1 h. IL-1β release into culture supernatants were analyzed by ELISA. Human THP-1 cells knockdowned by non-targeting siRNA pool were used as a negative control. The data were statistically analyzed by *t*-test and show the mean ±SD. n=3 per group. ***P*<0.01, ****P*<0.001 compared to the control THP-1 cells.

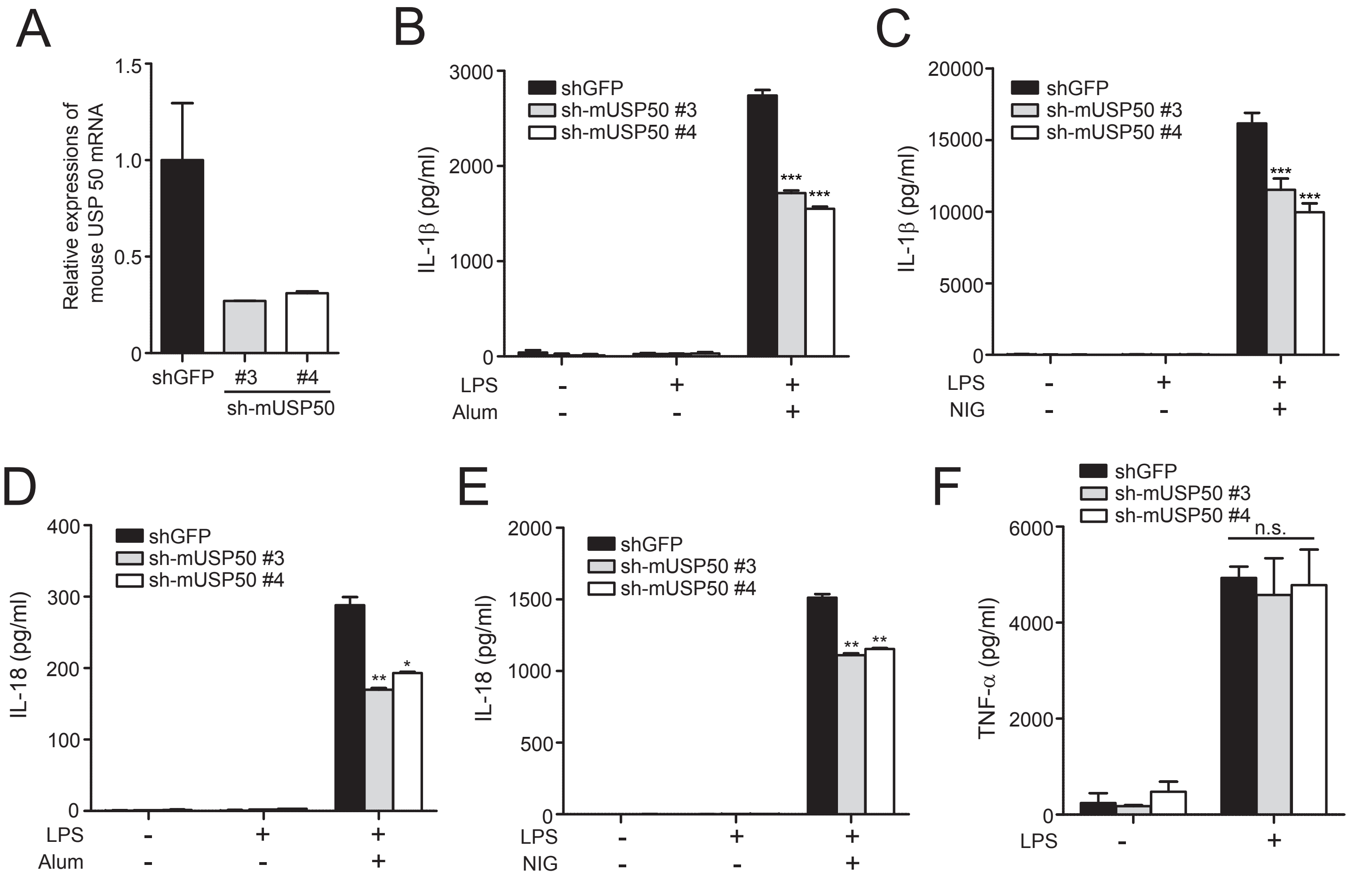

Supplementary Fig. 2. USP50 depletion in mouse BMDMs reduces NLRP3 inflammasome activation. (**A**) Mouse BMDMs were infected with concentrated lentiviruses expressing two independent shRNAs targeting mouse USP50 mRNA (sh-mUSP50 #3 and sh-mUSP50 #4). USP50 depletion was analyzed by real-time RT-PCR for USP50 transcripts. Lentiviruses expressing green fluorescence protein (GFP)-specific shRNA (shGFP) were used as a negative control. The data were statistically analyzed by a *t*-test and show the mean ± SD of three independent experiments. $^{**}P<0.01$ compared to control shGFP-expressing mouse BMDMs. (**B-E**) USP50-knockdowned BMDMs were treated with LPS (1 μg/ml) for 4 h, followed by Alum (300 μg/ml) for 6 h or NIG treatment (10 μM) for 1 h. Secretion of extracellular IL-1β (**B**, **C**) and IL-18 (**D**, **E**) into culture supernatants was analyzed by ELISA. (**F**) USP50-knockdowned or control BMDMs were treated with or without LPS. Secretion of extracellular TNF-α was analyzed by ELISA. All data in (**B-F**) were statistically analyzed by a *t*-test and show the mean ± SD. $^{*}P<0.05$, $^{**}P<0.01$, $^{***}P<0.001$ compared to the shGFP-expressing control BMDMs. n.s., not significant.

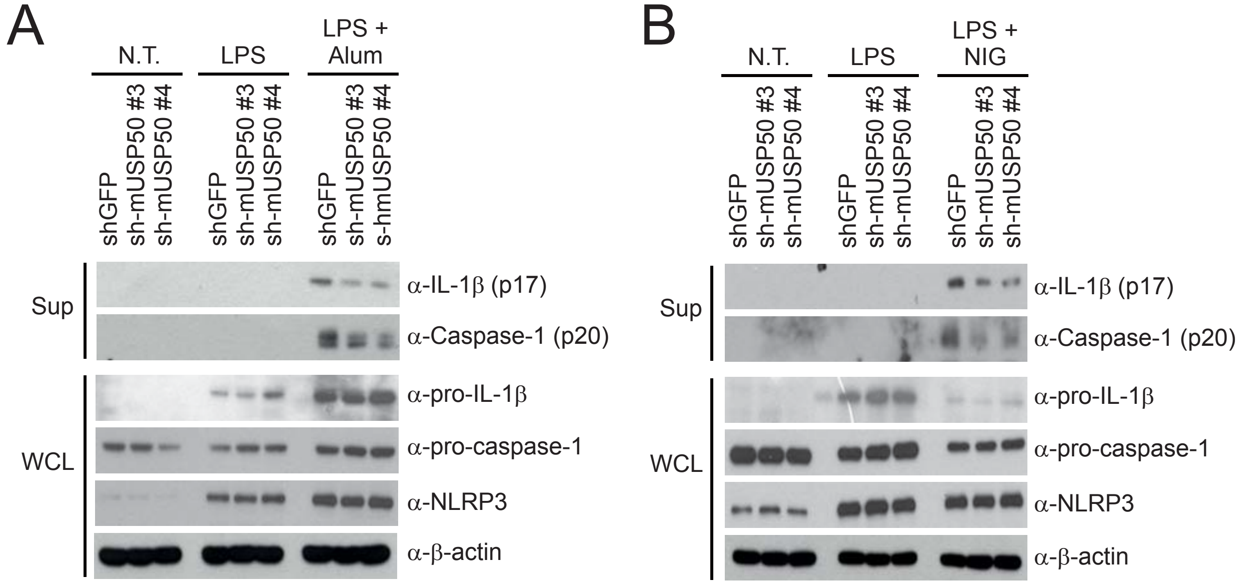

Supplementary Fig. 3. USP50 depletion in mouse BMDMs reduces the secretion of IL-1β.
(**A**, **B**) USP50-knockdowned BMDMs were treated with LPS, followed by Alum or NIG treatment under the same conditions as the experiments in Supplemnetary Fig. 2. Expression of active caspase-1 (p20) and the active form of IL-1β (p17) in culture supernatants (Sup) and whole cell lysates (WCL) was observed by immunoblot analysis. The data in (**A**) and (**B**) are representative of at least three independent experiments. shGFP-expressing mouse BMDMs were used as a negative control.

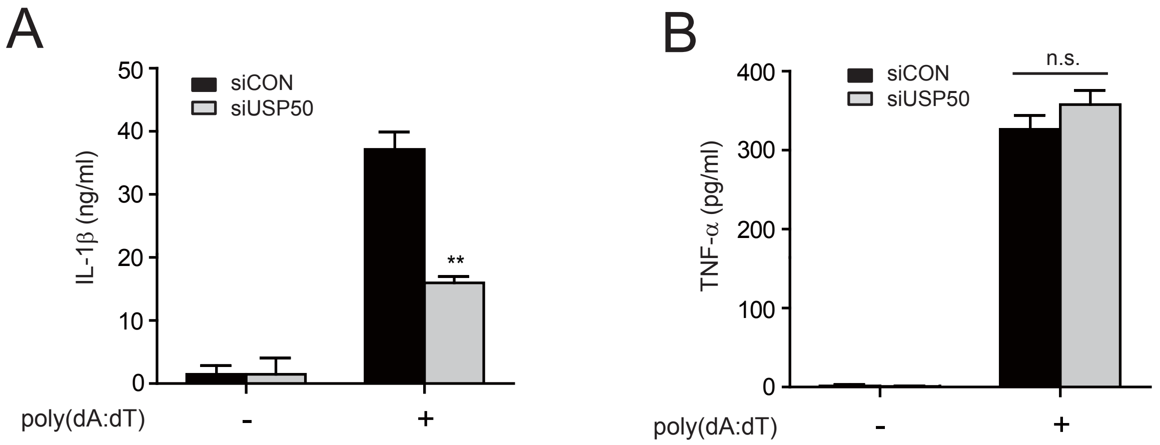

Supplementary Fig. 4. USP50 is required for activation of AIM2 inflammasome.
USP50-knockdowned THP-1 cells or control THP-1 cells were stimulated for 16 h after transfection of 1 μg/ml poly(dA:dT). Secretions of IL-1β (**A**) and TNF-α (**B**) into the culture supernatant were analyzed by ELISA. The data are representative of three independent replicates, statistically analyzed by a *t*-test and the mean ±SD is shown. ***P*<0.01compared to control THP-1 cells treated with AIM2 stimuli. n.s., not significant.

3. Supplementary Tables

Supplementary Table 1. Primer sequences used to construct plasmids in this study

Construct	**Direction**	**Sequence (5'- 3')**
Flag-USP50	Forward	ATGACTTCTCAGCCGTCTCT
	Reverse	TCAGGCCTGGGTGACTGA
HA-NLRP3	Forward	ATGAAGATGGCAAGCACCC
	Reverse	CTACCAAGAAGGCTCAAAGA
Flag-ASC, HA-ASC	Forward	ATGGGGCGCGCGCGCGAC
	Reverse	TCAGCTCCGCTCCAGGTCCT
HA-Pro-caspase-1	Forward	ATGGCCGACAAGGTCCTGA
	Reverse	TTAATGTCCTGGGAAGAGGTA
HA-Pro-IL-1β	Forward	ATGGCAACTGTTCCTGAA
	Reverse	TTAGGAAGACACGGATTC
Flag-USP50 (C53S)	Forward	CATTCACGCAGCTTGTGTTGCCCAAGT TCCACAAGC
	Reverse	GCTTGTGGAACTTGGGCAACACAAGCT GCGTGAATG
Flag-USP50 (H322A)	Forward	GGTGATTTGGATGGTGGCGCCTACACTG CTTTCTGCAA
	Reverse	TTGCAGAAAGCAGTGTAGGCGCCACCA TCCAAATCACC

Supplementary table 2. List of siRNA sequences used in this study

Lentiviral shRNA	Species	Target Sequences for USP50 depletion
shUSP50 #3	Mouse	TGACATCCAGGGCACAGTAAA
shUSP50 #4	Mouse	CCTGGACCTCACTCCTTATAT

siRNA	Species	Sequences
Si-NLRP3	Human	GCAAGACCAAGACGUGUGA
		GAAGUGGGGUUCAGAUAAU
		UGCAAGAUCUCUCAGCAAA
		GGAUCAAACUACUCUGUGA
Si-A20	Human	CUGCAGUACUUGCUUC
		CAACUCAUCUCAUCAAU
		UCUGGUAGAUGAUUAC
		CAACGAAUGCUUUCAG
Si-USP50	Human	UAUGAUACCCUUCCAG
		CAACACAUGCUGCGUG
		CUACCCAGCAUUUACGA
		GGACCUCACUCCUUAU
Non-targeting control	-	UGGUUUACAUGUCGACUAA
		UGGUUUACAUGUUGUGUGA
		UGGUUUACAUGUUUUCUGA
		UGGUUUACAUGUUUUCCUA

Supplementary Table 3. Primer for real-time RT-PCR used in this study

Construct	Direction	Sequence (5'- 3')
hUSP50	Forward	CCCATCAGCCTCCTTAACTG
	Reverse	AAATGACTTCTCAGCCGTCTC
mUSP50	Forward	CATTCACTCTTCTTCGGCAGT
	Reverse	GGCAGTCTTTACCCAGCTTT
hGapdh	Forward	TCATGACCACAGTCCATG
	Reverse	CACCACCTTCTTGATGTC
mGapdh	Forward	GTGGAGTCATACTGGAACATGTAG
	Reverse	AATGGTGAAGGTCGGTGTG
